# Effects of auditory training on children with developmental language disorder: a systematic review

**DOI:** 10.3389/fnhum.2025.1606860

**Published:** 2025-06-18

**Authors:** Wenxin Hu, Jialin Zhang, Tao Chen, Yuying Sun

**Affiliations:** ^1^Women’s Hospital of Nanjing Medical University, Nanjing Women and Children’s Healthcare Hospital, Nanjing, Jiangsu, China; ^2^State Key Laboratory of Reproductive Medicine and Offspring Health, Nanjing Medical University, Nanjing, Jiangsu, China; ^3^Tongji Hospital, School of Medicine, Tongji University, Shanghai, China; ^4^Shanghai Mental Health Center, Shanghai Jiao Tong University School of Medicine, Shanghai, China

**Keywords:** developmental language disorder, auditory training, language, auditory processing, systematic review

## Abstract

**Objective:**

This systematic review aims to evaluate the effectiveness of auditory training (AT) on various parameters, including language abilities, speech perception, auditory behavior, electrophysiological assessments, and working memory, in children with developmental language disorder (DLD) population.

**Methods:**

We searched PubMed, Web of Science, EMBASE, MEDLINE, Cochrane Library and CINAHL from inception to August 7, 2023, and further scrutinized the references of all selected articles. We included randomized controlled trials (RCTs) and quasi-experimental studies that investigated the effects of AT on children with DLD. Two researchers independently screened studies, extracted data and assessed risk of bias.

**Results:**

We included nine studies (eight RCTs and one quasi-experimental) in the systematic review, encompassing 379 children with DLD, 195 in the AT group and 184 in the control group. Compared to controls across five studies, AT did not significantly increase language abilities (expressive, receptive and total). Four out of five studies found significant improvements in children’s speech perception abilities after AT treatment, particularly phonological awareness and phoneme discrimination. Two studies showed improvements in temporal ordering and figure-context assessment, but two other studies found no significant changes. Two studies examining electrophysiological measures reported increased amplitudes in auditory event-related potentials after AT. Results for phonological working memory were inconsistent, with one study showing improvements in non-word repetition and digit span tasks, while another found no significant changes.

**Conclusion:**

Current evidence does not support the effectiveness of AT in enhancing core language abilities in children with DLD. However, AT may offer potential benefits for specific auditory processing skills and speech perception. More precise evaluation of the effectiveness of AT therapies in this population should be conducted in future research by employing rigorous methodology, bigger sample numbers and standardized outcome measures.

**Systematic review registration:**

www.crd.york.ac.uk/PROSPERO/myprospero, identifier CRD42024583480.

## What this paper adds?

Previous research on auditory training (AT) for children with developmental language disorder (DLD) has been limited by inconsistent methodologies, varying outcome measures and a lack of focus on long-term effects. In this study, we presented the first comprehensive systematic review dedicated to evaluating the effectiveness of AT interventions in this population. Our findings indicated that AT may have positive effects on auditory processing, speech perception and phonological working memory in children with DLD which can provide novel insights for early intervention strategies.

## 1 Introduction

Developmental language disorder (DLD) is a persistent neurodevelopmental condition affecting 6–15% of children, characterized by deficits in language acquisition, understanding, production and use (Bishop et al., 2017; World Health Organization, 2021). Without treatment, DLD can significantly impact academic performance, social interactions and future life prospects (Andreou and Aslanoglou, 2022; Graham et al., 2020; Duff et al., 2022; Gough Kenyon et al., 2020), potentially extending into adulthood (Goh et al., 2021; Dubois et al., 2020; Winstanley et al., 2021). While early interventions are crucial (Frizelle et al., 2023), current language therapies show limited long-term efficacy (Fan et al., 2022). Research priorities focus on evidence-based interventions for individual speech, language and communication goals in DLD (Kulkarni et al., 2022).

At present, there is no unified consensus on the neurobiological mechanism of DLD, but there are many theories that attempt to explain its potential causes, such as auditory temporal processing deficit (Protopapas, 2014), structural brain abnormalities in genetic (Whelan et al., 2018), neuroplasticity deficit (Price and Duman, 2020), auditory processing disorder (Ross-Swain, 2018) attention and executive function (Veríssimo et al., 2022). Auditory temporal processing deficit theory has received widespread attention in explaining DLD, and it can provide a measurable and neurally based cognitive mechanism explanation for the speech recognition, grammatical comprehension and language expression difficulties of DLD (Foss-Feig et al., 2017). A study suggests that not all children with DLD have temporal processing deficits, and some children’s language disorders are not accompanied by a low level of auditory temporal processing ability (Zapparrata et al., 2023). However, in the important cognitive and neural framework for understanding and intervening in DLD, temporal processing deficit theory can provide a theoretical framework to explain the neural mechanism-language symptoms-intervention path (Moll et al., 2016).

Auditory temporal processing deficit theory posits that children with DLD have difficulty processing rapidly unfolding auditory events, which affects their ability to discriminate speech (Szelag et al., 2015). The temporal sampling framework in the auditory temporal processing deficit theory links DLD to deficits in rise time perception and the ability to discriminate amplitude modulated sounds. The theory posits that children with DLD have difficulty perceiving and processing amplitude modulation signals, especially rhythms in the low frequency range (e.g., 2–50 Hz) (Goswami et al., 2016; Fraser et al., 2010; Martínez-Castilla et al., 2023).

In addition to the rise time perception and amplitude modulation discrimination disorders of the time sampling framework, DLD is also associated with other auditory processing (AP) defects, such as audition discrimination deficit, audition sequencing defects, auditory working/short-term memory defects and abnormal dichotic Listening (de Wit et al., 2018; Jones et al., 2024) AP mainly involves the perceptual processing of auditory information in the central auditory nervous system (CANS) which generates electrophysiological auditory potentials through neurobiological activities (ASHA, 2005). A study comparing the language abilities of children with DLD and normal children of the same age found that children with DLD performed poorly in speech recognition and speech contrast tasks, indicating that children with DLD have difficulty distinguishing phonemes which hinders the acquisition of phonological rules (Ziegler et al., 2005). Moreover, many studies have shown that DLD children perform poorly compared to children of the same age in perceiving and remembering the sequence of a series of sounds, maintaining and manipulating speech information, understanding speech in incomplete or disrupted situations, integrating information from the left and right ears into a unified perception (Blom and Boerma, 2020; Moran, 2022; Singer et al., 2023).

Given that children with DLD may have defects in AP, researchers have begun to try to use auditory training (AT) as an intervention to improve their language function (Rinaldi et al., 2021). Studies have shown that AT can improve speech recognition, auditory memory and language fluency in certain groups of language disorders, especially the phonological awareness and speech recognition ability of children with DLD (Chermak and Musiek, 2013). Since children with DLD show high heterogeneity in auditory processing, it is very important to choose the appropriate AT intervention method. There are currently two main methods for AT intervention. Current studies mostly use non-linguistic auditory stimuli (such as pure tones and frequency changes) or speech elements without semantic load (such as meaningless syllables) as training materials to isolate the influence of language processing and focus on the underlying perceptual mechanism (McArthur et al., 2008). However, some studies think that incorporating language components into the training paradigm can improve its ecological validity and better translate into benefits in real language use scenarios (Fey et al., 2011).

Many studies have applied AT to treat children with DLD, but its therapeutic effect has always been controversial. A comprehensive review by Murphy and Schochat (2013) analyzed the effects of various types of auditory timing training on language skills (Murphy and Schochat, 2013). The study found that AT has potential benefits for certain language skills, but further research is needed for the DLD population. Although this study analyzed the effects of AT on disorders related to language difficulties, there are relatively few studies on the effects of AT on the DLD population. Therefore, this study evaluated the intervention effects of two AT intervention models on DLD children by synthesizing existing research, which provides a theoretical basis for further understanding the potential mechanisms of DLD and formulating more targeted intervention strategies.

## 2 Materials and methods

We reported the findings The findings are reported in accordance with the Preferred Reporting Items for Systematic Reviews and Meta-Analyses (PRISMA) guidelines (Page et al., 2021). The protocol for this systematic review was registered with PROSPERO, registration number CRD42024583480.

### 2.1 Data sources and searches

We systematically searched the following electronic databases from inception to August 7, 2023: PubMed, Web of Science, MEDLINE, CINAHL, Embase, Cochrane Central Register of Controlled Trials, and Cochrane Database of Systematic Reviews in the Cochrane Library.

We developed a comprehensive search strategy using a combination of Medical Subject Headings (MeSH) terms and free-text keywords related to the population (e.g., “child*,” “adolescent*,” “preschool”), condition (e.g., “DLD,” “SLI,” “language disorder*”), intervention (e.g., “auditory,” “train*”), and study design (e.g., “random*”, “control*”). We tailored the search strategy for each database, and a full electronic search strategy for one database is provided in Supplementary material 1.

### 2.2 Eligibility criteria

We included studies meeting the following criteria: (1) participants: children and adolescents (aged < 18 years) diagnosed with DLD according to the International Classification of Diseases 11th Revision (ICD-11) criteria or equivalent diagnostic standards; (2) intervention: comparative studies involving AT alongside academic enrichment, speech and language therapy, a waitlist, or no treatment; (3) primary outcomes: use standardized/norm referenced test measure various aspects of language abilities, such as overall language proficiency, language expression and reception in the areas of semantics, syntax, phonology and narration; secondary outcomes: auditory processing ability, speech perception, and working memory; (4) study design: both randomized clinical trials (RCTs) and quasi-experimental studies were considered; (5) language of publication: English. Given that the definition of DLD has evolved, studies were included if their definition of DLD aligned with the current international classification of DLD (ICD-11). ICD-11 is the latest version released by the World Health Organization (WHO) in 2018. Exclusion criteria were as follows: (1) unavailability of full text or original data; (2) duplicate publications.; (3) non-intervention studies, such as case reports, case series, cross-sectional studies, qualitative studies, review articles, expert opinions, editorials and studies based on animal models; (4) non-peer-reviewed literature, such as conference abstracts, dissertations, book chapters and gray literature.

### 2.3 Study selection

All the literature was imported into EndNote, and then articles with duplicate titles and authors were deleted. Two independent reviewers screened titles and abstracts of all retrieved records against the eligibility criteria. We obtained full-text articles for all potentially eligible studies, which two reviewers then independently assessed for inclusion. We resolved disagreements through discussion or, if necessary, consultation with a third reviewer.

### 2.4 Data extraction

We developed a standardized, pre-piloted form to extract data from the included studies. Two reviewers independently extracted the following information: (1) study characteristics: first author, publication year, country, study design, diagnostic criteria; (2) participant characteristics: sample size, age range, gender distribution, ethnicity (if reported), socioeconomic status (if reported); (3) intervention details: type of AT, duration, frequency, comparison intervention;(4) outcome measures: instruments used, time points of measurement; (5) results: means, standard deviations, effect sizes (where available).

### 2.5 Risk of bias assessment

We Two independent researchers assessed the risk of bias in the included RCTs using the Cochrane Risk of Bias 2 (RoB 2) tool (Sterne et al., 2019), this tool assessed five domains: (1) bias arising from the randomization process; (2) bias due to deviations from intended interventions; (3) bias due to missing outcome data; (4) bias in the measurement of the outcome; (5) bias in the selection of the reported result. If any domain presented either some concern or a high risk of bias, the overall risk for the RCT was rated as either “some concern” or “high risk.” Otherwise, the RCT was classified as “low risk.” For quasi-experimental studies, we utilized the Risk of Bias in Non-randomized Studies of Interventions (ROBINS-I) tool (Sterne et al., 2016), which assesses seven domains of bias and rated the overall risk as either “low risk,” “moderate risk,” or “serious/critical risk.”

Two reviewers independently assessed risk of bias. In case of disagreement, we resolved it through discussion or consultation with a third reviewer. We summarized the risk of bias assessment results using the Risk of Bias Visualization Tool (ROBVIS) (McGuinness and Higgins, 2021). If all answers did not indicate a potential problem, the domain was considered to have a low risk of bias. Conversely, a “serious risk” in any domain means that the effect estimate of the study is seriously affected.

### 2.6 Data synthesis

Given the anticipated heterogeneity in interventions and outcome measures, we conducted a narrative synthesis of the findings. We grouped studies by outcome category and intervention characteristics. Within each group, we summarized the direction and magnitude of effects, considering the strength of evidence based on study design, sample size and risk of bias.

## 3 Results

### 3.1 Study selection

**FIGURE 1:**
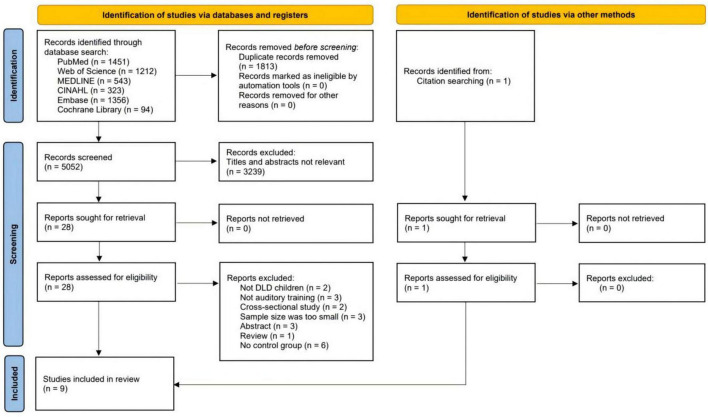
PRISMA 2020 flow diagram for new systematic reviews.

Figure 1 presented the PRISMA flow diagram illustrating our study selection process. The initial database search yielded 5,052 records. After removing 1,813 duplicates, 3,239 records remained for screening. Based on title and abstract review, 3,211 studies were excluded. We then assessed the full text of 28 articles for eligibility, of which eight met the inclusion criteria. An additional study was identified through citation searching, a total of nine studies included in the final systematic review.

### 3.2 Characteristics of included studies

We included nine studies in this review: eight RCTs and one quasi-experimental study. As detailed in Table 1, these studies involved. As detailed in Table 1, these studies involved a total of 379 participants, with individual study sample sizes ranging from 14 to 108 participants. All studies reported participant ages, which ranged from 4 to 13 years. Six studies reported participant gender, with males comprising 63.6% of the sample. The studies were conducted between 2005 and 2019, across various countries: United States (*n* = 2), the United Kingdom (*n* = 2), Poland (*n* = 2), Germany (*n* = 1), Belgium (*n* = 1), and Brazil (*n* = 1).

**TABLE 1:** Characteristics of included studies.

References (country)	Design	Age (Mean ± SD, years)	Sample Size (T/C)	Sex (M/F)	Experimental Intervention	Control intervention	Frequency	Duration (weeks)	Post-intervention evaluation	Outcome assessments
Cohen et al. (2005) (United Kingdom)	RCT	T: 7.34 ± 1.29; C: 7.43 ± 1.21	T: 23; C: 27	T: 16/7; C: 17/10	FFW-L	Untrained	90 mins/d, 5 d/week	6	9 weeks and 6 months after baseline	Receptive, Expressive, and Total Language (CELF-3); Vocabulary (TOLD-P:3); Grammatic Understanding; Phonological Assessment
Bishop et al. (2006) (United Kingdom)	RCT	T: 10.85 ± 1.79/ 11.08 ± 1.13; C: 10.28 ± 0.88	T: 24; C: 9	−	Group 1: Slow Speech training; Group 2: Modified Speech training	Untrained	20 sessions, 15 mins/session	12	12 weeks after baseline	Grammar (TROG-2); Narrative language (ERRNI); Non-speech auditory assessments; Speech auditory assessments
Gillam et al. (2008) (United States)	RCT	T: 7.42; C: 7.58	T: 54; C: 54	T: 29/25; C: 35/19	FFW-L	Academic enrichment	100 mins/d, 5 d/week	6	Immediately, 3 months, 6 months after training	Expressive and receptive language (CASL); Backward Masking; Sentence comprehension (Token Test for Children); Phonological awareness (C-TOPP)
Fey et al. (2010) (United States)	RCT	T: 7.41 ± 0.51; C: 7.57 ± 0.61	T: 7; C: 9	−	FFW-L	Untrained	24 sessions, 100 mins/session	6	Immediately after training	Narrative language (NLAI); Grammar test; Phonological working memory (Non-word Repetition Test)
Collet et al. (2012) (Belgium)	RCT	T: 8.75; C: 8.58	T: 9; C: 9	T: 7/2; C: 6/3	Adaptive auditory discrimination training	Untrained	18 sessions, 20 mins/session	4	During and immediately after training, 1 month after	Phonological awareness; Vocabulary task
Szymaszek et al. (2018) (Poland)	RCT	T: 6.4 ± 0.9; C: 6.0 ± 0.9	T: 14; C: 13	T: 9/5; C: 10/3	TIP training	Non-TIP training (speech therapy)	1 h/session, 3 sessions/week	8	Immediately after training	Phonetic identification (VOT task); Phoneme Discrimination Test; TIP (auditory temporal order threshold)
Dacewicz et al. (2018) (Poland)	RCT	T: 6.3 ± 1.0; C: 6.0 ± 0.8	T: 18; C: 18	T: 13/5; C: 13/5	TIP training	Non-TIP training (speech therapy)	1 h/session, 3 sessions/week	8	Immediately after training	Electrophysiological assessment (MMN; N2; N2’; P3a)
Roden et al. (2019) (Germany)	RCT	T: 4.52 ± 0.64; C: 4.51 ± 0.56	T: 40; C: 37	T: 24/16; C: 22/15	Auditory stimulation training	Untrained	30 mins/session, 3 sessions/week	12	Immediately after training	Phonological working memory (Digit Span Test, Non-word Recall Test, Recall of Sentences Test); Phoneme Discrimination Test; Speech Perception Test
Filippini et al. (2012) (Brazil)	Quasi-experimental	9.09 ± 1.54	T: 6; C: 8	−	AT + speech therapy	Speech therapy	1 time/week, 50 mins/time	8	4 weeks after training	Auditory Behavioral Assessment (Speech-in-Noise test, Staggered Spondaic Word test, Dichotic Digits test, Pitch Pattern Sequencing test); Electrophysiological Assessment (c-ABR)

NS, T: training group; C, control group; M, male; F, female; RCT, randomized clinical trial; FFW-L, Fast ForWord-Language; AT, auditory training; TIP, temporal information processing; CELP-3, The Clinical Evaluation of Language Fundamentals—Third Edition UK; TOLD-P:3, The Test of Language Development-Primary, Third Edition; BAS II Word Reading Scale: The British Ability Scales: Second Edition Word Reading Scale; TROG-2, Test for Reception of Grammar-2; ERRNI, Expression, reception and recall of narrative instrument; CASL, comprehensive assessment of spoken language; C-TOPP, Comprehensive Test of Phonological Processing; NLAI, Narrative Language Ability Index; VOT, Voice-onset-time.

AT interventions varied considerably across studies. The studies by Dacewicz et al. (2018); Szymaszek et al. (2018) and Filippini et al. (2012) clearly conducted AP baseline assessments and designed targeted intervention programs based on the assessment results (Supplementary Table 1). Other studies mainly relied on theoretical assumptions and lacked assessments of participants’ specific AP deficits. Four studies focused on verbal auditory interventions, three on non-verbal auditory interventions, and two incorporated both verbal and non-verbal auditory interventions. The duration of AT interventions ranged from 4 to12 weeks.

### 3.3 Risk of bias assessment results

**FIGURE 2:**
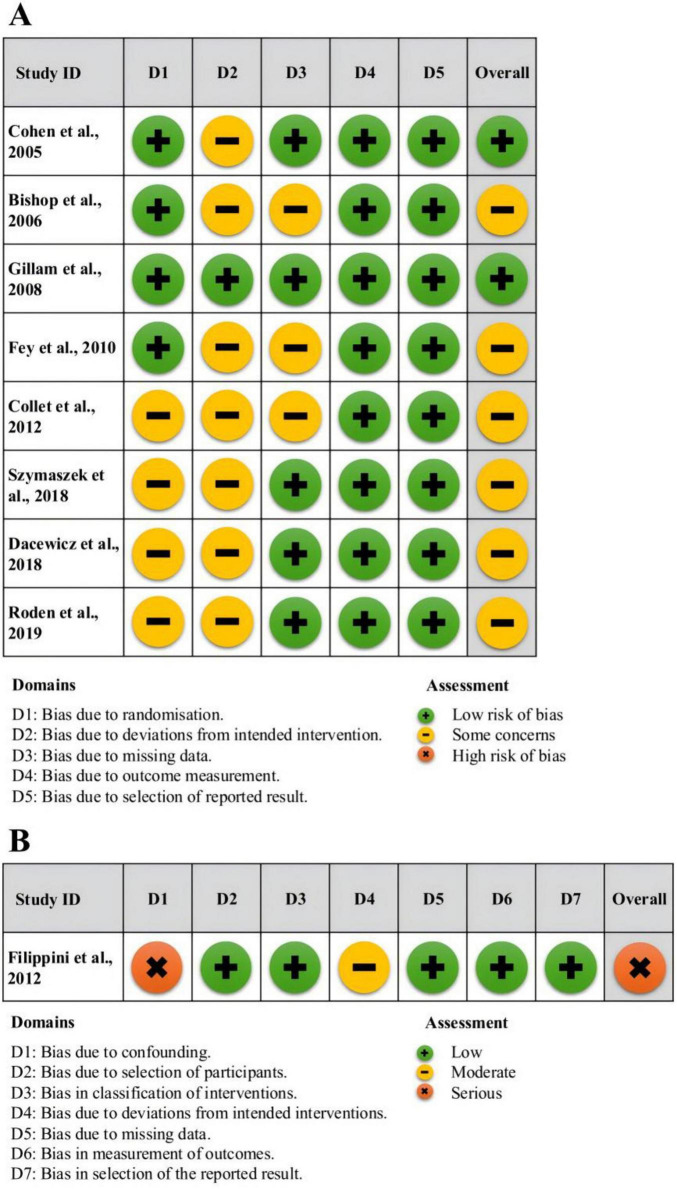
Results of risk of bias assessment.

Among the eight RCTs, two were assessed as having a low overall risk of bias, while six were found to have some concerns, as outlined in Figure 2. Four RCTs were evaluated as having a low risk of bias due to the randomization process, whereas the rest had some concerns in this domain. Most randomized controlled trials (*n* = 7, 88%) exhibited some concerns regarding bias due to deviations from the intended interventions; only one study was rated as low risk in this domain. We considered deviations from intended interventions when assessing risk of bias. For example, studies that reported significant protocol changes during the intervention period or had issues with intervention adherence were flagged as having some concerns in this domain. Studies that maintained strict adherence to the intervention protocol and reported no significant deviations were assessed as low risk. Five studies were assessed as having a low risk of bias due to missing outcome data, while three exhibited low risk in this aspect. All RCTs were deemed to have a low risk of bias in measurement of outcomes and selection of reported results. Overall, 25% of the included RCTs were assessed as having a low risk of bias, and approximately 75% had some concerns. The quasi-experimental study was determined to have a serious risk of bias, primarily attributed to confounding factors.

### 3.4 Effects of auditory training

Our analysis of the included studies shows conflicting results about the efficacy of AT therapies for children with DLD. Overall, we found that AT interventions showed limited effectiveness in improving core language skills. However, some studies reported improvements in specific areas such as speech perception and certain aspects of auditory processing. The inconsistency in results across studies may be attributed to the heterogeneity in intervention methods, outcome measures and study designs. We categorized the outcomes into five main areas, language skills, speech perception, behavioral auditory processing, auditory electrophysiological assessment and phonological working memory.

#### 3.4.1 Language skills

Five studies investigated the effects of AT on language skills (Bishop et al., 2006; Cohen et al., 2005; Collet et al., 2012; Fey et al., 2010; Gillam et al., 2008) with three utilizing the FFW-L program as the intervention method (Cohen et al., 2005; Fey et al., 2010; Gillam et al., 2008). Studies by Cohen et al. (2005) and Gillam et al. (2008) specifically explored the effects of the FFW-L program on both expressive and receptive language abilities. These studies found no significant differences in either receptive or expressive language skills between the intervention group and either the control group or academic enrichment groups that received no intervention. Cohen et al. (2005) revealed that the overall language abilities in the AT group did not significantly differ from those of the untrained control group following FFW-L-based training. Bishop et al. (2006) and Fey et al. (2010) focused on the effects of AT on narrative language and also found no significant improvement in narrative language capabilities when compared to untrained control groups. Cohen et al. (2005); Bishop et al. (2006) and Fey et al. (2010) reported on grammar-related outcomes, showing no significant improvements in the grammar skills of the children undergoing AT compared to untrained controls. Gillam et al. (2008) corroborated these findings, noting that FFW-L did not significantly improve sentence comprehension compared to an untrained control group. Furthermore, Collet et al. (2012) found that adaptive auditory discrimination training did not improve vocabulary scores across sessions.

#### 3.4.2 Speech perception

Five studies reported different results regarding the speech perception (Cohen et al., 2005; Collet et al., 2012; Gillam et al., 2008; Roden et al., 2019; Szymaszek et al., 2018). Gillam et al. (2008) and Collet et al. (2012) found that AT resulted in significant improvements in children’s phonological awareness when compared to both a control group without any intervention and an academic enrichment group. Szymaszek et al. (2018) and Roden et al. (2019) focused on the capacity to differentiate between phonemes which is a feature of phonological awareness. The intervention group showed notable improvements in this area. In contrast, there were no significant improvements found in either the non-auditory temporal processing intervention or the control group that did not receive any training. Furthermore, Szymaszek et al. (2018) reported improvements in phonetic identification ability following AT. However, Cohen et al. (2005) found no significant difference in phonological assessment ability between AT and untrained control groups after the post-intervention.

#### 3.4.3 Behavioral auditory processing

Four studies investigated the effects of AT on behavioral assessments of auditory processing in children, yielding inconsistent conclusions and employing diverse assessment tools (Bishop et al., 2006; Filippini et al., 2012; Gillam et al., 2008; Szymaszek et al., 2018). Bishop et al. (2006) investigated the effects of slow speech training or modified speech training on both non-verbal (frequency and duration threshold tests) and verbal auditory processing skills (speech discrimination in quiet and in noise). Their results indicated no significant improvements in either speech or non-speech discrimination skills within the intervention group. Gillam et al. (2008) found no significant enhancement in the backward masking abilities of children with DLD when participating in the FFW-L program compared to an academic enrichment group. In contrast, Szymaszek et al. (2018) reported that auditory temporal information processing training (TIP) led to a significant improvement in auditory temporal thresholds. Filippini et al. (2012) also found statistically significant improvements in temporal sequencing and figure-ground assessments within the intervention group following AT, improvements not reported in children who did not undergo AT.

#### 3.4.4 Auditory electrophysiological assessment

Two studies investigated changes in electrophysiological assessments (Dacewicz et al., 2018; Filippini et al., 2012). Dacewicz et al. (2018) investigated MMN, N2, N2’ and P3 amplitude and latency changes following auditory temporal processing training. The results indicated that in the TIP group, post-test measurements revealed increased MMN, which was accompanied by enhanced N2 and N2’ amplitudes for deviant stimuli, a pattern not evident in the non-TIP control group. Furthermore, MMN amplitude was higher in the TIP group compared to the control group after the intervention. Although both groups displayed higher P3a amplitudes and shorter latencies in the post-test, no significant differences in P3 amplitude were found. Filippini et al. (2012) used c-ABR as an outcome measure and found that children with DLD who received both AT and speech therapy showed significantly greater improvements in peaks V, C, D, and E at the final assessment compared to those who received speech therapy alone.

#### 3.4.5 Phonological working memory

Two studies investigated outcomes related to phonological working memory (Fey et al., 2010; Roden et al., 2019). Fey et al. (2010), and reported no statistically significant changes in non-word repetition test scores for children with DLD after undergoing the FFW-L program intervention. Conversely, Roden et al. (2019) found an significant improvement in the non-word repetition skills among children with DLD who participated in auditory stimulation training. Roden et al. (2019) also assessed digit span test and sentence memory performance pre- and post-intervention, revealing a significant enhancement in working memory capacity within the auditory stimulation training cohort.

## 4 Discussion

This systematic review aimed to investigate the effects of AT on children diagnosed with DLD. Our analysis of nine studies (Eight RCTs and one quasi-experimental study) revealed mixed results regarding the efficacy of AT for this population. The inconsistent findings across various domains of language and auditory processing underscore the heterogeneous nature of DLD and the challenges in developing universally effective interventions.

Murphy and Schochat’s study mainly explored the potential promotion of three types of auditory time training (software-assisted training, formal auditory training, and music training) on children’s language ability (Murphy and Schochat, 2013). The evaluation index mainly focused on language skills themselves. This review specifically evaluates the effect of auditory training in children with DLD, including five aspects: language ability, speech perception, auditory behavior, electrophysiological response, and speech working memory. The results showed that although the training did not significantly improve core language skills, it may have a positive effect in improving phonetic awareness and certain auditory processing abilities.

Our review found limited evidence for the efficacy of AT in improving core language skills in children with DLD. Five studies consistently reported no significant improvements in various aspects of language abilities following AT interventions (Bishop et al., 2006; Cohen et al., 2005; Collet et al., 2012; Fey et al., 2010; Gillam et al., 2008). This finding contrasts with previous research on aphasia, which has demonstrated neuronal reorganization and enhanced synaptic activity in language-related brain areas following AT (Woodhead et al., 2017). For individuals with more severe conditions, enhanced interhemispheric transfer of information was observed between higher levels of the auditory cortex. However, our findings diverge from previous studies. This discrepancy may be attributed to the heterogeneous nature and broad spectrum of clinical symptoms within the DLD population (Toppelberg and Shapiro, 2000), suggesting that auditory processing deficits alone may not fully account for the language impairments observed in this population. Another limitation is that existing research on the efficacy of AT for language skills in children with DLD focuses predominantly on school-aged children (4–13 years in our included studies), thereby missing the timeframe of peak brain plasticity (Cardon et al., 2012). This could explain the lackluster impact of AT on language outcomes. Furthermore, three of the included studies employed the FFW-L program for their training modules (Cohen et al., 2005; Fey et al., 2010; Gillam et al., 2008). Meta-analyses from 2011 suggest that FFW-L does not yield significant benefits in any measured outcomes, such as single-word reading, passage reading comprehension, receptive language, and expressive language, when compared to active or untreated control groups (Strong et al., 2011). This factor may also have influenced the outcomes of our review, indicating a need for future studies to employ a broader range of AT approaches to fully explore language outcomes in DLD.

Despite the limited impact on overall language abilities, our review found more promising results in specific domains. For speech perception, five of the studies included in this review investigated the topic of speech perception, with four demonstrating significant improvements in speech perception skills following AT. This aligns with prior research indicating that individuals in the DLD group consistently underperform across various aspects of phonological awareness (Lara-Díaz et al., 2021). The improvements observed in speech perception suggest that AT may have potential benefits for foundational language skills, even if these improvements do not immediately translate to broader language abilities. For phonological awareness, our review revealed inconsistent results with previous research, which has established that phonetic awareness skills are strong predictors of future reading capabilities in children (Song et al., 2019). Therefore, early AT is anticipated to bolster the phonological awareness of children with DLD, consequently mitigating the risk of developing dyslexia.

For auditory processing measures, our review revealed inconsistent results. Two studies utilizing FFW-L and modified speech training did not demonstrate significant improvements in auditory behavioral tasks. These findings cast doubt on the claims that FFW-L could effectively address rapid auditory processing deficits in children with language impairment (Corporation, 1999). Additionally, the data provided little support for the effectiveness of modified speech training, and no correlation was observed between the number of training sessions and outcomes. However, two other studies in the review demonstrated that AT significantly improved temporal sequencing ability, figure-ground assessment, and auditory temporal thresholds. These results align well with previous research, suggesting that AT is effective in ameliorating auditory processing deficits (Loo et al., 2016). Future research in this field should focus on designing appropriate training regimens to further investigate whether enhancements in auditory processing skills can lead to corresponding improvements in language abilities.

In the studies included in this systematic review, the dosage of AT varied significantly, the duration of training ranged from a few weeks to several months, the duration of a single training session ranged from 30 min to 2 h, and the frequency of training ranged from twice a week to every day. This high heterogeneity may explain the inconsistency of the research results to some extent. Some studies have found that high-frequency and long-duration interventions are more effective in improving phonological awareness, while no significant changes were observed with lower-dose training (Harvey, 2022; Deng et al., 2024). However, some studies have not observed significant improvements in core language abilities even with higher-dose interventions, indicating that the dosage of AT is not the only factor that determines the efficacy (Justice, 2018; Storkel et al., 2019). Some studies believe that the content of training, individual differences in children, and the stage of language development at the time of intervention are also very important for children with DLD (Eisenberg, 2014; Cummings et al., 2021).

Electrophysiological measures provided interesting insights into the potential neural mechanisms underlying AT’s effects. Two studies reported significant increases in the amplitudes of MMN, P3, and c-ABR following AT. These findings suggest that AT may induce neurophysiological changes, even in the absence of clear behavioral improvements. This aligns with prior research utilizing magnetic resonance imaging (MRI) to evaluate brain plasticity following auditory cognitive training in older individuals has also supported these observations (Kawata et al., 2022). Specifically, the auditory cognitive training group exhibited improvements in pure-tone audiometry and showed increases in both regional gray matter volume and functional connectivity (FC) within the left temporal pole, in comparison to a control group not undergoing auditory cognitive training (Kawata et al., 2022). These results lend credence to the notion that AT may offer considerable benefits for neural function.

Previous findings have demonstrated that children with DLD exhibit poorer verbal and non-verbal working memory performance is poorer in children with DLD compared to their TD counterparts (Larson and Ellis Weismer, 2022). Our review examined two studies that investigating the effects of AT on phonological working memory, revealing inconsistent outcomes. While the FFW-L intervention did not lead to a significant enhancement in phonological working memory in school-aged children, research by Roden et al. demonstrated significant improvements in performance on the digit span, non-word recall, and recall of sentences tests, when compared to a control group. These results align with earlier work indicating that typically developing children can improve their auditory working memory through music training (Lee et al., 2007; Roden et al., 2014). The current study adds to this literature by suggesting that AT may also benefit the phonological short-term memory and phonological coding strategies for written words in children with DLD. One explanation for the discrepancy between the two studies could be the age of the participants during the intervention.

The age range of children is a key factor in designing AT, and the neurodevelopment of the central auditory system varies significantly at different ages (Chermak and Musiek, 2002). Early neuroplasticity provides a favorable time window for intervention, but young children have limited abilities in attention, self-regulation and task execution which may affect the realization of training effects. In addition, children of different ages also differ in the use of auditory processing strategies and cognitive resources (Herzberg et al., 2025). Although some studies have shown that the efficacy of AT for children with DLD is limited, there is evidence that AT may bring positive intervention effects in a variety of communication disorders, such as speech perception disorders, SLI and auditory processing disorders (Harvey, 2022; Deng et al., 2024).

The preschool years are associated with greater brain plasticity compared to the school-age years. This is especially pertinent when considering that deficits in phonological working memory often persist in children with delayed language development, even after receiving successful speech therapy (McGonigle-Chalmers and Kusel, 2019). Therefore, it is necessary to comprehensively consider the developmental stage and individual differences of children to optimize the training effect and enhance language-related functions in clinical applications.

## 5 Limitations

The current review has several limitations that warrant discussion. First, despite our comprehensive search strategy, which included six major databases and additional manual searching, we only included articles published in English. This language restriction may have introduced a potential bias, as relevant studies published in other languages could have been overlooked. However, given the rigorous nature of our review process and the consistency of our findings across the included studies, the main conclusions of our study would not significantly change even if non-English publications were included. Second, many of the studies were conducted at single centers and featured small sample sizes. In particular, some studies included fewer than ten DLD cases. Second, many of the studies were conducted at single centers and featured small sample sizes. In particular, some studies included fewer than ten DLD cases (Collet et al., 2012; Fey et al., 2010; Filippini et al., 2012), which could compromise the reliability of the overall findings. Lastly, the overall methodological quality of the included studies was suboptimal, Our risk of bias assessment revealed that out of the eight RCTs, only two were assessed as having a low overall risk of bias, while six had some concerns. The quasi-experimental study included in our review was determined to have a serious risk of bias. Fast ForWord is a multifaceted intervention rather than a strictly defined auditory training (AT) approach. FFW combines auditory, language, and cognitive training modules, so the improvements observed in the study cannot be clearly attributed to auditory training alone, but may also be the result of language or cognitive training. These methodological limitations underscore the need for higher-quality research with more rigorous study designs to robustly assess the effects of AT on children with DLD.

## 6 Conclusion

This systematic review investigates the effects of AT for children with DLD, suggesting limited efficacy in improving overall language abilities, but demonstrates potential in enhancing specific skills like speech perception and phonological awareness. Electrophysiological evidence suggests possible neurophysiological changes. The heterogeneous outcomes underscore DLD’s complexity and the need for individualized interventions. Clinically, AT should be considered as an adjunctive treatment. Future research should focus on large-scale studies with standardized protocols, examining long-term outcomes and efficacy across diverse linguistic contexts, to develop evidence-based interventions for DLD.

## Data Availability

The original contributions presented in the study are included in the article/Supplementary material, further inquiries can be directed to the corresponding author.

## Supplementary material

Supplementary Material 1 Search strategy.

Supplementary Table 1 Auditory training (AT) intervention methods used in studies on children with DLD.
