# Supplementary material for: Effects of auditory training on children with developmental language disorder: a systematic review
Source: Front Hum Neurosci. 2025 Jun 18;19:1606860. doi: 10.3389/fnhum.2025.1606860 (PMC12213595; doi:10.3389/fnhum.2025.1606860)
Supplement: Supplementary Material 1 — Search strategy. [file Data_Sheet_1.docx]

**Supplement 1. Search Strategy**

**Pubmed**

Search date: August 7, 2023

Results: 1474

(auditory) AND ("therapy" [Subheading] OR "Therapeutics"[Mesh] OR therap* OR intervention OR train*) AND (("Child"[Mesh] OR "Child, Preschool"[Mesh] OR "Adolescent"[Mesh]) OR (child* OR adolescent*)) AND (("Language Disorders"[Mesh] OR "Specific Language Disorder"[Mesh] OR "Language Development Disorders"[Mesh] OR "Specific Language Impairment 4" [Supplementary Concept]) OR ("language disorder*" OR "language impairment*" OR "language deficit*" OR "language development disorder*" OR DLD OR SLI OR "language delay" OR "language retardation"))

**Web of Science**

Search date: August 7, 2023

Results: 1212

(auditory) AND ("Education" OR "therap*" OR "Therapeutics" OR therapy OR intervention OR train*) AND (child* OR adolescent* OR preschool*) AND ("language disorder*" OR "language impairment*" OR "language deficit*" OR "language development disorder*" OR "language delay" OR "language retardation" OR DLD OR SLI)

**CINAHL**

Search date: August 7, 2023

Results: 323

("auditory") AND ((MH "Child+") OR "child*" OR (MH "Child, Preschool") OR (MH "Adolescent") OR "adolescent*" OR "preschool*") AND ((MH "Specific Language Disorder") OR (MH "Language Development Disorders+") OR (MH "Language Disorders+") OR ("language disorder*") OR ("language impairment*") OR ("language deficit*") OR ("language delay") OR ("language retardation") OR ("SLI") OR ("DLD")) AND (train* OR intervention OR therapy OR educat*)

**MEDLINE**

Search date: August 7, 2023

Results: 543

("auditory") AND ((MH "Child+") OR "child*" OR (MH "Child, Preschool") OR (MH "Adolescent") OR "adolescent*" OR "preschool*") AND ((MH "Specific Language Disorder") OR (MH "Language Development Disorders+") OR (MH "Language Disorders+") OR ("language disorder*") OR ("language impairment*") OR ("language deficit*") OR ("language delay") OR ("language retardation") OR ("SLI") OR ("DLD")) AND (train* OR intervention OR therapy OR educat*)

**EMBASE**

Search date: August 7, 2023

Results: 1356

('child'/exp OR 'preschool child'/exp OR 'adolescent'/exp OR 'preschool*' OR 'child*' OR 'adolescent*') AND ('language disability'/exp OR 'developmental language disorder'/exp OR 'language disorder*' OR 'language impairment*' OR 'language deficit*' OR 'language difficul*' OR 'languauge development disorder*' OR 'dld' OR 'sli' OR ‘language delay’ OR ‘language retardation’) AND (**auditory**) AND (**'training'**/exp OR **'therapy'**/exp OR **therap*** OR **educat***)

**Cochrane**

Search date: August 7, 2023

Results: 94

#1 MeSH descriptor: [Child] explode all trees

#2 MeSH descriptor: [Adolescent] explode all trees

#3 MeSH descriptor: [Child, Preschool] explode all trees

#4 #1 OR #2 OR #3 OR child* OR adolescent* OR preschool*

#5 MeSH descriptor: [Language Disorders] explode all trees

#6 MeSH descriptor: [Specific Language Disorder] explode all trees

#7 MeSH descriptor: [Language Development Disorders] explode all trees

#8 #5 OR #6 OR #7 OR (language NEXT disorder*) OR (language NEXT impairment*) OR (language NEXT deficit*) OR (language NEXT development NEXT disorder*) OR DLD OR SLI OR ("language delay") OR ("language retardation")

#9 auditory

#10 MeSH descriptor: [Therapeutics] explode all trees

#11 #10 OR therap* OR training OR educat* OR intervention

#12 #4 AND #8 AND #9 AND #11
